# Supplementary material for: Influence of the grounding zone on the internal structure of ice shelves
Source: Nat Commun. 2025 May 12;16:4383. doi: 10.1038/s41467-025-58973-2 (PMC12069554; doi:10.1038/s41467-025-58973-2)
Supplement: Supplementary file 1 — Supplementary Information [file 41467_2025_58973_MOESM1_ESM.pdf]

Supplementary material for:

Influence of the grounding zone on the internal structure of ice shelves

K. E. Miles*, B. Hubbard, A. Luckman, B. Kulessa, S. Bevan, S. Thompson, and G. Jones

* Corresponding author: k.miles1@lancaster.ac.uk

Supplementary Table 1: Eigen analysis summary information. Primary eigenvalues and the strike and dip of the primary eigenvector for each unit at each site. Strike is relative to true North.

Site	Unit	Primary eigenvalue	Strike (°)	Dip (°)
JP-21	1	0.97	23.3	1.1
	2	0.94	41.6	51.3
	3	0.94	41.6	28.0
	4	0.91	50.2	76.2
SI-47	1	0.99	68.5	2.4
	2	0.76	222.6	2.5
	4	0.97	36.3	63.0

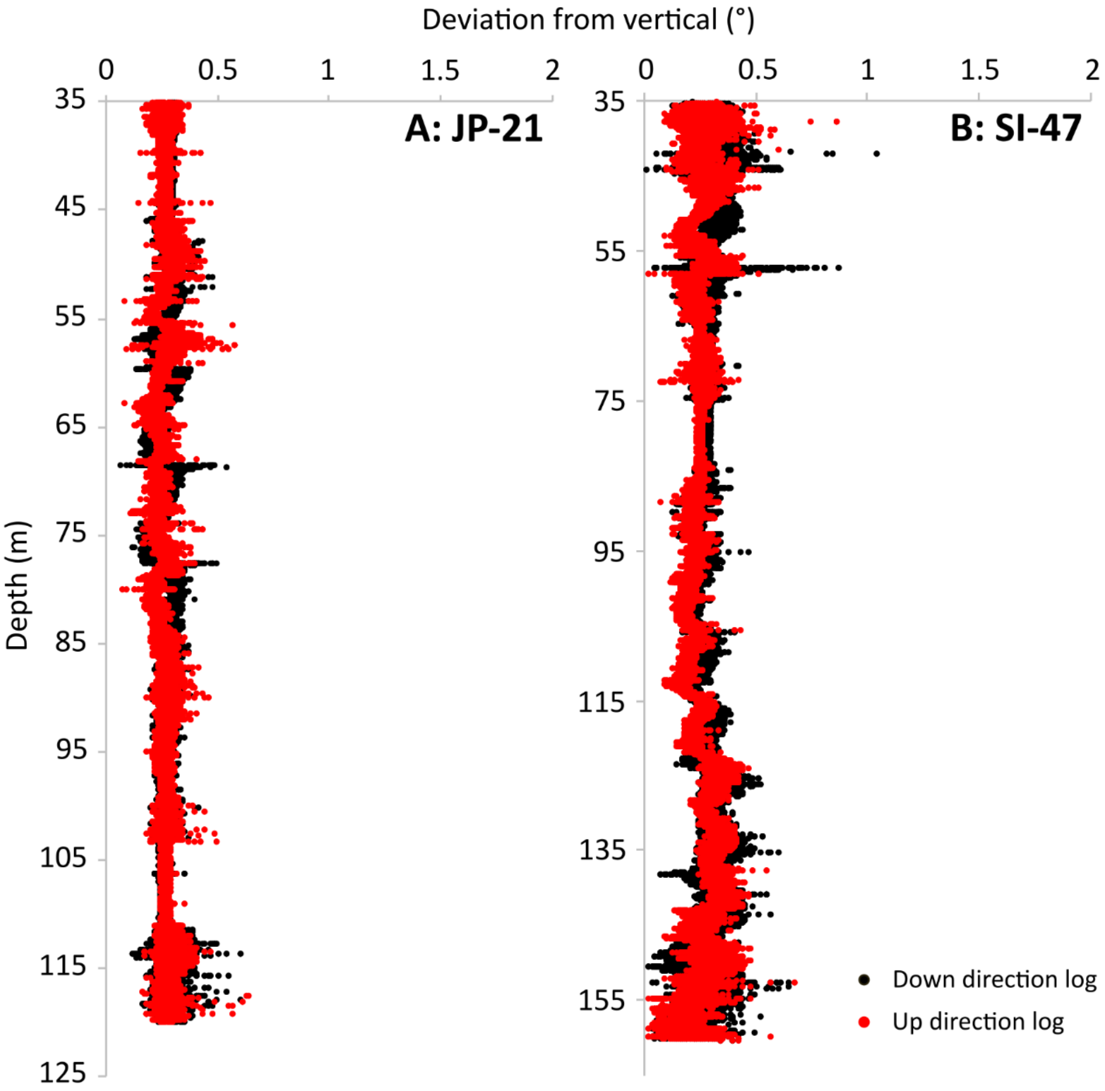

Supplementary Figure 1: Deviation of each borehole's inclination from vertical, measured by the optical televiewer, for JP-21 (**A**) and SI-47 (**B**). Borehole dip is only plotted below borehole water level because the record is degraded by the probe wobbling in the uppermost air-filled sections. Source data are provided in the source data file.

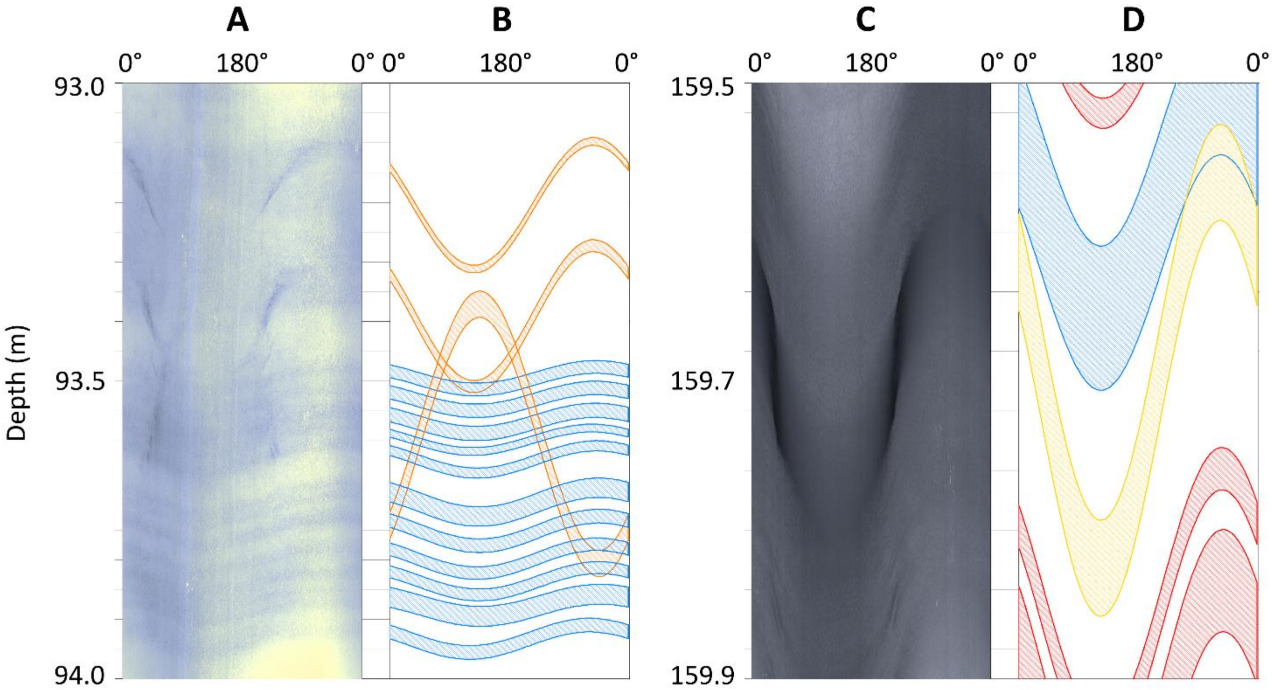

Supplementary Figure 2: Optical televiewer logs and delineated layers at depth, for a section of borehole JP-21 (**A** and **B**) and SI-47 (**C** and **D**). Logs were recorded moving up the borehole, are orientated to true North, and shown here in true colour. Layer colours on the structural logs of delineated layers (panels B and D) reflect the initial categorising of layers, which was later refined to: red – ice layers; orange and yellow –bubble-free ice layers (crevasse traces); and blue – faint ice layers.

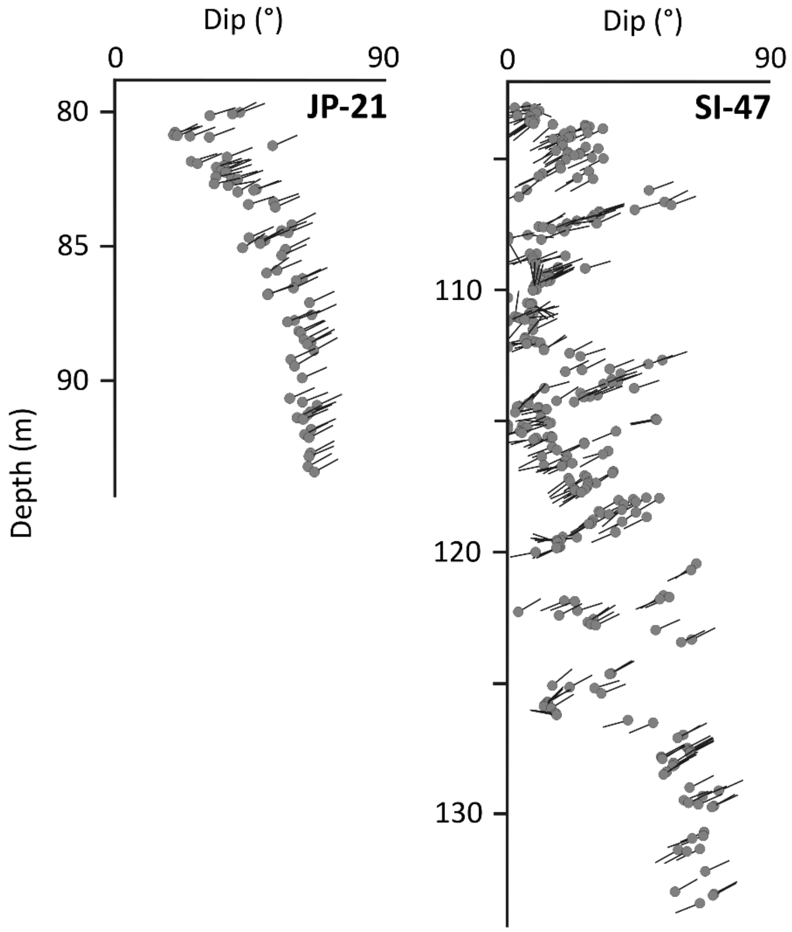

Supplementary Figure 3: Tadpole plots for all delineated layers in Unit 2 for each borehole. The dip of each layer is indicated by the grey circles, with values read off the x-axis. The strike of each layer is plotted by the tail of each circle as the angle relative to Antarctic Stereographic Polar North, so as to be directly comparable to Figure 5 in the main manuscript. Source data are provided in the source data file.

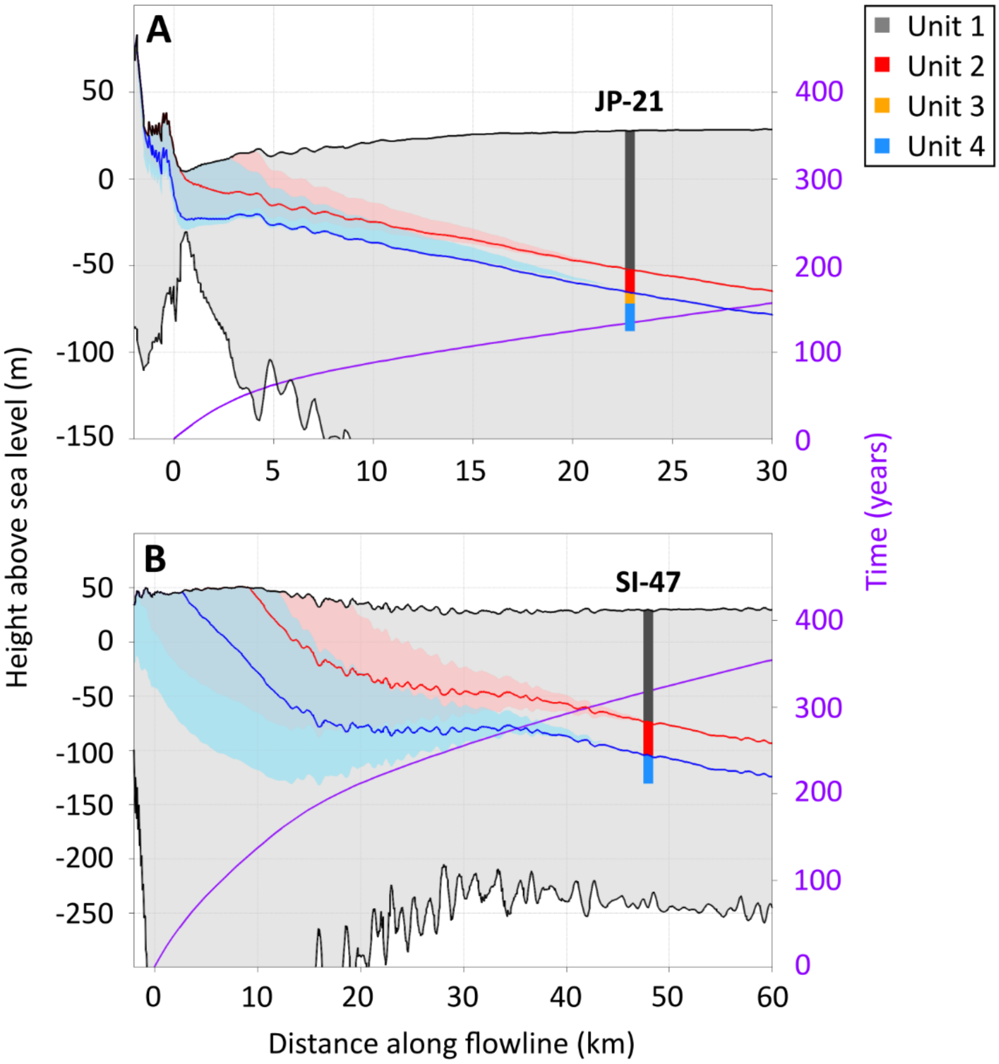

Supplementary Figure 4: Reverse flowline model outputs of the location of the upper (red line) and lower (blue line) boundaries of Unit 2 from the borehole sites towards the grounding zone for A) JP-21, and **B)** SI-47. Boreholes are coloured by unit (following Figure 3 in the main manuscript). The median model estimate of the upper and lower boundaries of Unit 2 is shown by the red and blue lines, respectively (see Methods). The uncertainty bounds are shown by light shaded regions: red for the upper boundary of Unit 2 are the 10–90th percentiles for JP-21 and minimum–maximum surface mass balance values for SI-47; blue for the lower boundary of Unit 2 are the 25–75th percentiles for JP-21 and minimum–maximum surface mass balance values for SI-47 (see Methods). These vary because the flowline modelling for JP-21 could not start high enough that the same uncertainty bounds (min-max as used in the Figure 6) could be used for both sites. Time in years since ice crossed the grounding zone is shown by the secondary purple axis and line. Upper and lower ice surfaces are taken from MEaSUREs Bedmachine Antarctica V.3. Source data are provided in the source data file.

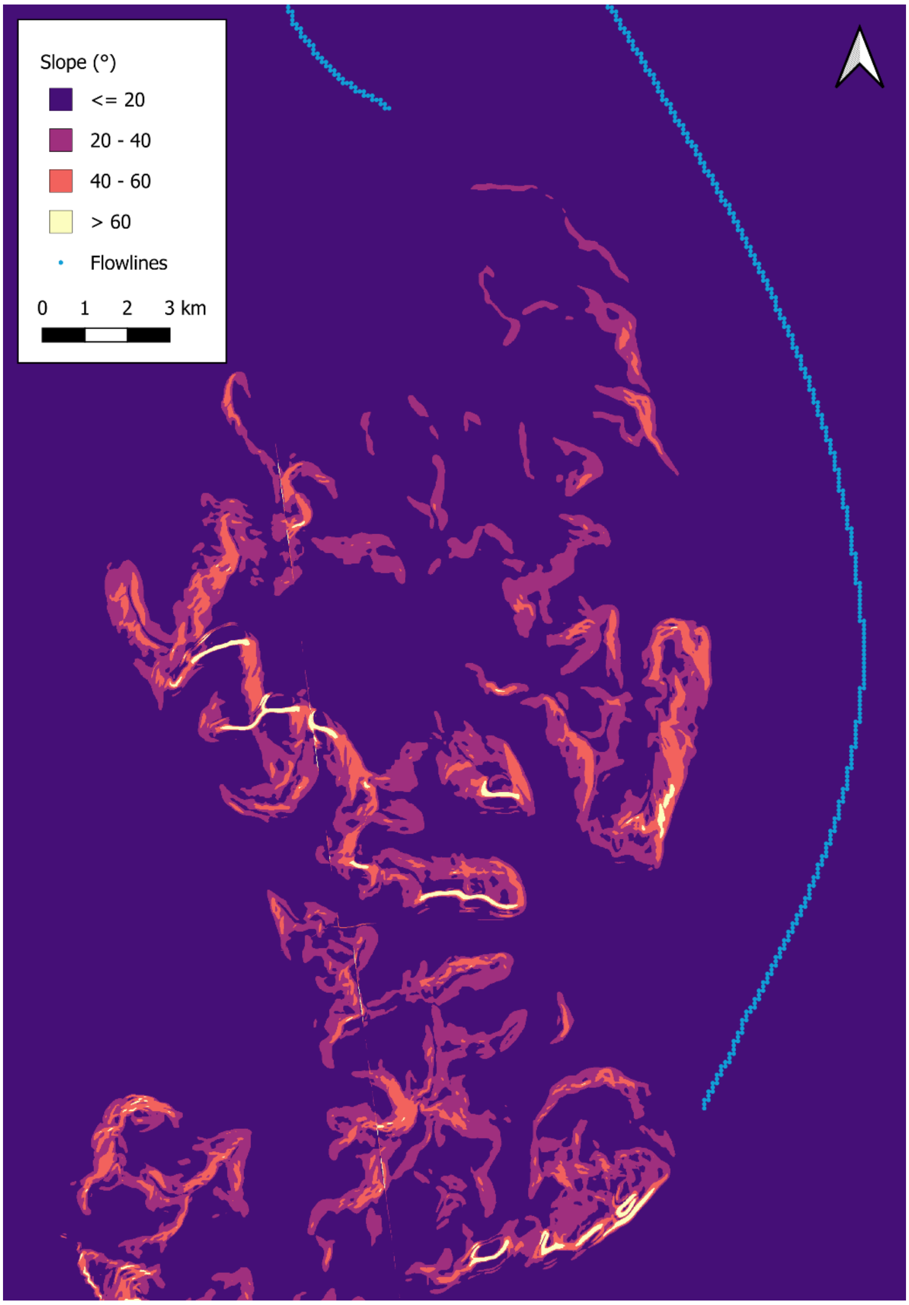

Supplementary Figure 5: Surface slope over the Joerg Peninsula, calculated from the TanDEM-X DEM (from interferometry using scenes from 2012 and 2022).

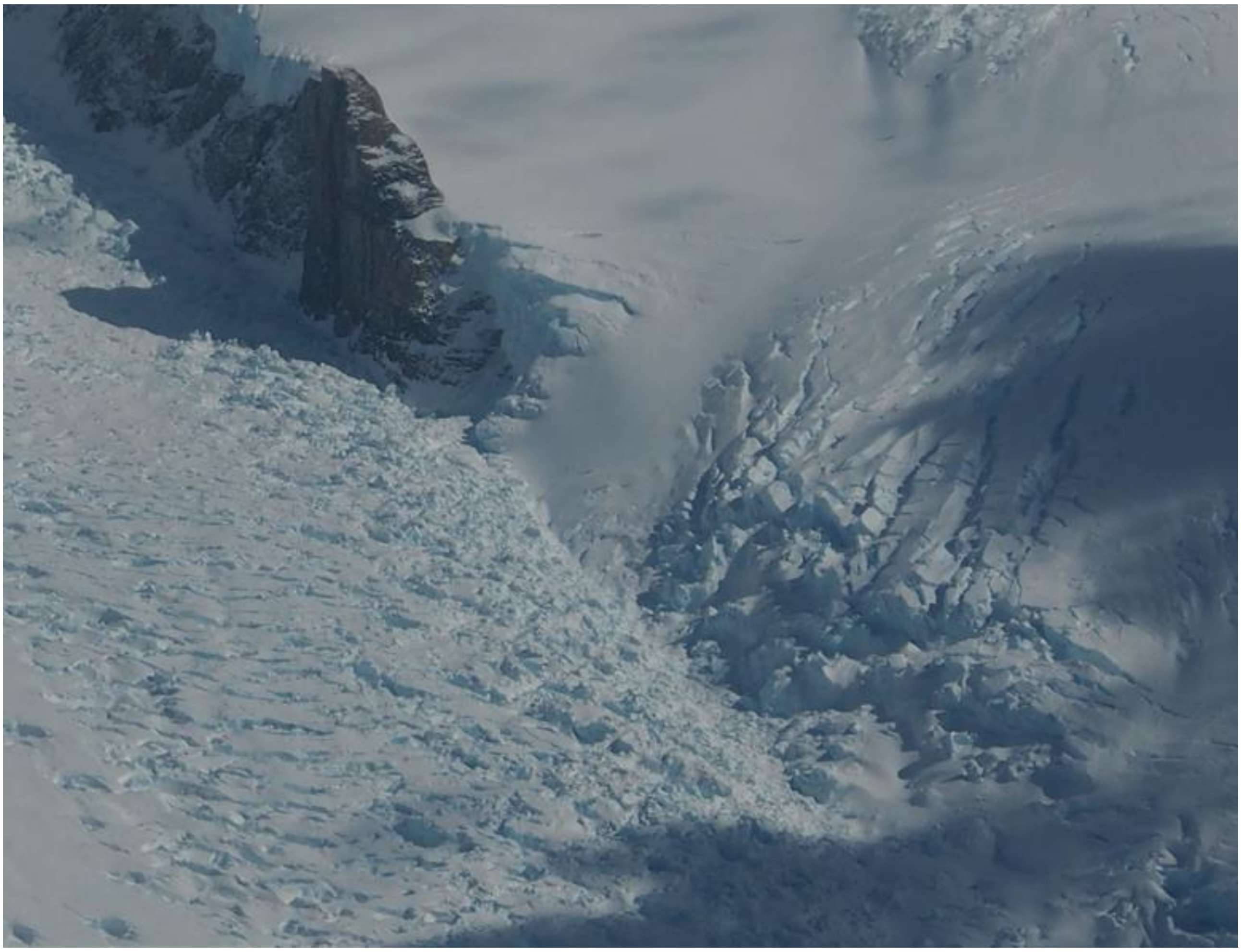

Supplementary Figure 6: Aerial image of intense deformation as a grounded glacier flows over the grounding zone in the north of Larsen C Ice Shelf.

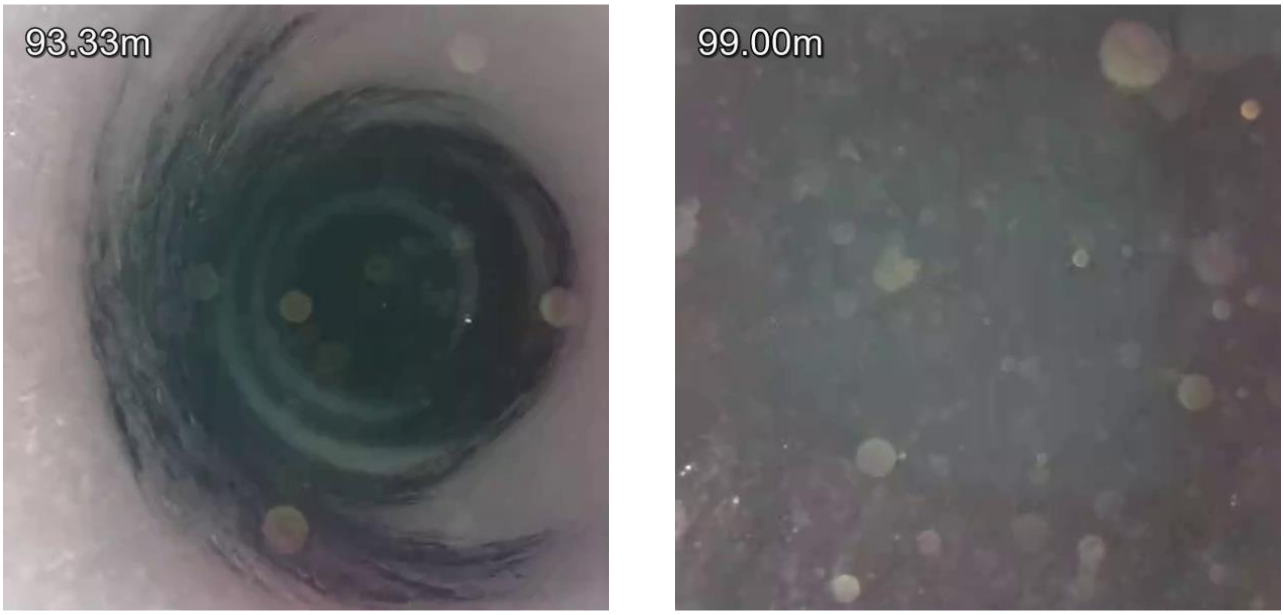

Supplementary Figure 7: Screenshots from a down-borehole-viewing video camera deployed immediately after the optical televiewer at JP-21. The image taken at 93.33 m depth shows a few individual ice platelets floating in the borehole. The image taken at 99 m depth shows the borehole full of platelets, obscuring the borehole wall.
